# Challenges of Population-based Measurement of Suicide Prevention Activities Across Multiple Health Systems

**DOI:** 10.5334/egems.277

**Published:** 2019-04-12

**Authors:** Bobbi Jo H. Yarborough, Brian K. Ahmedani, Jennifer M. Boggs, Arne Beck, Karen J. Coleman, Stacy Sterling, Michael Schoenbaum, Julie Goldstein-Grumet, Gregory E. Simon

**Affiliations:** 1 Kaiser Permanente Northwest, Center for Health Research, Portland, OR, US; 2 Henry Ford Health System, Center for Health Policy and Health Services Research, Detroit, MI, US; 3 Kaiser Permanente Colorado Institute for Health Research, Aurora, CO, US; 4 Kaiser Permanente Southern California, Department of Research and Evaluation, Pasadena, CA, US; 5 Kaiser Permanente Northern California, Division of Research, Oakland, CA, US; 6 National Institute of Mental Health, Division of Services and Intervention Research, Bethesda, MD, US; 7 Zero Suicide Institute, Education Development Center, Washington DC, US; 8 Kaiser Permanente Washington, Health Research Institute, Seattle, WA, US

**Keywords:** suicide prevention, health systems, electronic health record, population-based, zero suicide

## Abstract

Suicide is a preventable public health problem. Zero Suicide (ZS) is a suicide prevention framework currently being evaluated by Mental Health Research Network investigators embedded in six Health Care Systems Research Network (HCSRN) member health systems implementing ZS. This paper describes ongoing collaboration to develop population-based process improvement metrics for use in, and comparison across, these and other health systems. Robust process improvement metrics are sorely needed by the hundreds of health systems across the country preparing to implement their own best practices in suicide care. Here we articulate three examples of challenges in using health system data to assess suicide prevention activities, each in ascending order of complexity: 1) Mapping and reconciling different versions of suicide risk assessment instruments across health systems; 2) Deciding what should count as adequate suicide prevention follow-up care and how to count it in different health systems with different care processes; and 3) Trying to determine whether a safety planning discussion took place between a clinician and a patient, and if so, what actually happened. To develop broadly applicable metrics, we have advocated for standardization of care processes and their documentation, encouraged standardized screening tools and urged they be recorded as discrete electronic health record (EHR) variables, and engaged with our clinical partners and health system data architects to identify all relevant care processes and the ways they are recorded in the EHR so we are not systematically missing important data. Serving as embedded research partners in our local ZS implementation teams has facilitated this work.

## Context

The age-adjusted suicide rate in the United States increased 28 percent between 1999 and 2016 when nearly 45,000 Americans died by suicide [1], a figure that is likely an underestimate given the high burden of proof on coroners and medical examiners to determine that suicide deaths are intentional [2]. Though it is a leading cause of death in the United States, suicide is a preventable public health problem [3].

In 2012, the National Action Alliance for Suicide Prevention (Action Alliance) and the U.S. Surgeon General published the National Strategy for Suicide Prevention (NSSP) [4]. A call to action, the NSSP outlines a comprehensive, long-term plan to address the heavy burden of suicide in the U.S. Among its top four priorities are the integration of suicide prevention into health care reform and the transformation of health care delivery systems to significantly reduce suicide morbidity and mortality. More recently, the Action Alliance issued recommended standard care guidelines for people with suicide risk [5]. These guidelines advance goals 8 and 9 of the NSSP: To promote suicide prevention as a core component of health care services (Objective 8) and to promote and implement effective clinical and professional practices for assessing and treating those identified as at-risk for suicidal behaviors (Objective 9) [4]. Health care settings are recognized as one of the most promising environments to implement suicide prevention practices. Indeed, among patients at least minimally engaged in health care, most make an outpatient visit in the year prior to their suicide death, almost half have a visit within a month of their death [6], and nearly all of those who make non-fatal suicide attempts have received at least some prior year outpatient care [7]. Each of these visits are opportunities for suicide prevention.

The NSSP promotes the adoption of “zero suicides” as an aspirational goal for health systems serving defined populations (Objective 8.1) [4]. One systems-level suicide prevention framework, Zero Suicide (ZS) [8], is currently being promoted by the Substance Abuse and Mental Health Services Administration (SAMHSA) and is widely implemented throughout the United States and internationally [9]. ZS is a flexible set of evidence-based interventions, recommendations, and strategies encompassing seven domains (lead, train, identify, engage, treat, transition, improve) that, collectively, are designed to mitigate suicide risks, enhance protective factors, and close gaps in health care that leave at-risk patients vulnerable. ZS implementation begins with strong leadership commitment to systemwide change that promotes suicide prevention as a core responsibility, followed by strategic planning, training, and practice changes. Specifically, resources available for implementation include a free and publicly available toolkit (www.zerosuicide.com), an active e-list with over 2000 members, a workforce survey, and an organizational self-study. Implementation strategies are meant to be tailored to each health system’s unique population and local context. Therefore, ZS is not a one-size-fits-all, manualized intervention. Rather, it is a set of recommendations, best practices and customizable tools to improve the quality of suicide prevention efforts.

ZS promotes several evidence-based suicide prevention interventions developed in the past decade and is based on the Henry Ford Health System (HFHS) Perfect Depression Care program that resulted in a near 80 percent reduction in the suicide death rate among patients receiving behavioral health care [10,11,12,13,14], an impressive rate that was sustained for more than a decade [12,13,15]. Since then, other health systems implementing ZS have also measured and observed reductions in suicide mortality [16]. However, apart from these few systems, no large-scale evaluation of ZS has been conducted and therefore implementation endorsements are moving in advance of robust evidence to support the model. More rigorous study is needed to understand suicide outcomes within various health systems, service settings (e.g., primary care), and diverse patient populations. Further, it is unclear which specific ZS components, bundle of components, or process of care implementation strategies are most effective.

## Case description

Currently, investigators within the Mental Health Research Network (MHRN) [17] are conducting an evaluation of the implementation of ZS across six health care systems serving more than nine million patients annually. The MHRN is able to accurately capture suicide attempt and death outcomes because each system has a defined patient population with comprehensive electronic health records (EHRs) and insurance claims data available to track health care use within and outside of each participating system. Data includes complete capture of injury or poisoning diagnoses from all care settings (ambulatory care, emergency department, and inpatient). The MHRN has also linked official government mortality records to health system records at each site to measure suicide death. This is unique as most health systems do not consistently link their populations to mortality data.

Because of their relationships to the health systems and their roles as embedded researchers, the MHRN investigators are also able to accurately identify and describe suicide care processes and measure quality improvements. The health systems participate together in a Zero Suicide national learning collaborative where they share approaches, decisions, and lessons learned as they design a unique implementation strategy for their individual systems. The systems are also working together with MHRN researchers to compare and evaluate specific components of ZS. Health system and clinical leaders with responsibility for ZS implementation and health system data analysts are partners in the research; the project aims to demonstrate a ‘Learning Healthcare System for Suicide Prevention.’

Because each of the health systems are implementing different ZS components in different settings (e.g., behavioral health, primary care) at different times, certain sites will serve as ‘intervention’ sites for specific ZS components while others serve as ‘controls’ allowing a pragmatic interrupted time-series analysis of suicide outcomes that overcomes the costs and impracticalities of a randomized controlled trial. This kind of evaluation, using EHR data to measure ZS processes and outcomes across multiple diverse health systems implementing varied suicide prevention approaches within defined, large populations sufficiently powered to test suicide outcomes, could only be conducted within the HCSRN. One practical goal of this project is to develop EHR-based tools for implementation and evaluation that can be replicated in other health systems using EHR data.

This paper describes the work our team is doing to develop population-based suicide prevention metrics for use in, and comparison across, the health systems. While measuring suicide outcomes is important and comes with its own unique set of challenges [18,19], here we discuss measuring process improvements. Robust process improvement metrics are necessary for the evaluation study but are also sorely needed by the hundreds of health systems across the country preparing to implement their own best practices in suicide care, as well as by other entities and organizations implementing ZS through funded federal awards from SAMHSA, the National Institute of Mental Health, the Indian Health Service and others. Developing generalizable EHR-based suicide prevention metrics is complicated. Here we articulate three examples of challenges in using health system data to assess suicide prevention activities, each in ascending order of difficulty. These challenges are not unique to this evaluation study, nor to suicide prevention research, and represent broader difficulties encountered when using EHR data to assess processes and outcomes across health systems, even among systems with enriched data resources such as those in the HCSRN.

## Findings

### Challenge 1: Mapping and reconciling different versions of suicide risk assessment instruments across health systems

ZS emphasizes the importance of using an evidence-based, scorable risk assessment tool. For the purposes of population risk management, ideally that tool would be easily be embedded in the EHR with discrete data fields, so the data can be easily identified and harvested for quality improvement monitoring. Most health systems participating in the ZS evaluation study use the Patient Health Questionnaire (PHQ-9) [20] item 9 to identify potential suicide risk and have been doing so, as standard care, within the EHR, for some time. The PHQ-9 is a discrete, standardized tool to screen for depression; it has a single suicide item (item 9) which is used clinically to determine which patients might require additional assessment of suicide risk [21]. While versions of the PHQ-9 have varied slightly across and within the participating health systems and over time, because of its long history of use in these systems for both care delivery and research, it is relatively easy to determine whether a PHQ-9 was collected. PHQ-9 scores (summary and item-level) are easily harvested from EHR data at most of these systems; the MHRN has used PHQ-9 data extensively in our suicide prevention research.

In contrast, the Columbia Suicide Severity Rating Scale (C-SSRS) [22], used to assess suicide ideation severity and intensity and suicide behavior, also a discrete, standardized tool, has only recently been made available in the EHR to all participating health systems. It has not been fully implemented in all systems or in a standardized manner across systems. For example, some systems plan to eventually use the C-SSRS exclusively in all departments throughout the health system while others are only using some of the items and only in certain settings (e.g., behavioral health department). Modes of C-SSRS administration have also varied (e.g., electronic and paper versions). In some systems scores have been recorded as discrete data fields but more frequently scores, or sometimes only a narrative summary, have been documented in progress notes. This variability increases the likelihood of undercounting important risk assessment efforts and creates much greater difficulty in operationalizing use of the C-SSRS for measuring suicide prevention. Ongoing work with the C-SSRS is focused on standardizing metrics and data collection across sites for use in quality improvement efforts and research.

### Challenge 2: Deciding what should count as adequate suicide prevention follow-up care and how to count it in different health systems with different care processes

Despite the aforementioned challenges, suicide risk screening involves a relatively standardized care process, unlike follow-up care once risk is identified, which could include many different interventions at varying levels of care with distinct clinicians. Considerable effort has been made to coordinate staff, organize workflows, and bolster services that will address additional risk uncovered by more comprehensive risk screening. Many health systems have developed monitoring and outreach programs but the way those programs are organized and how patient contacts are documented varies widely across health systems, making measurement of risk mitigation exceedingly complicated. There is also the issue of whether health systems are implementing core ZS components with fidelity. Local adaptation is necessary as long as it does not compromise fidelity. Both measuring how ZS is implemented and what is being implemented are important quality improvement goals. One challenge for developing generalizable suicide prevention metrics is determining what efforts should be measured; a secondary challenge is determining how those efforts can be measured using EHR data across different systems.

Returning to the example of our systems’ relative advantage of using easily retrievable PHQ-9 data, if a health system process improvement initiative is, for example, to administer the PHQ-9 at all mental health specialty visits for patients 13 and older, the measurement question is straightforward: “How often is a PHQ-9 recorded during these visits?” In systems with robust capture of well-organized PHQ-9 data this is an uncomplicated metric to produce. However, if a follow-up care process improvement initiative is to administer the C-SSRS for all mental health specialty patients scoring 2 or 3 on PHQ-9 item 9, and the measurement question is “How often is the C-SSRS recorded during these visits?” then it becomes necessary to identify all sources of C-SSRS scores (see above) and specify what counts as a qualifying C-SSRS. For example, if the clinician administers the C-SSRS but records the result in the narrative section of the progress note only, rather than in the C-SSRS flowsheet (where the data can be easily retrieved), will that C-SSRS score be captured and counted? Further, if the first C-SSRS item, “Have you wished you were dead or wished you could go to sleep and not wake up?” is not endorsed by the patient and no further questions are administered, does that C-SSRS administration still count?

As another example, if a process improvement initiative is to schedule a follow-up visit within two weeks for every patient scoring ≥3 on the C-SSRS, should any subsequent visit count? Does the visit need to be in-person or would video visits, phone encounters, or secure message exchanges also satisfy the requirement? Does the visit need to be with the same clinician? Should the visit be with a behavioral health clinician or would a visit with an accompanying diagnostic code in any department by any clinician count? Does there need to be some kind of documentation that suicide risk was acknowledged? If the intent of the visit is to follow up on the elevated suicide risk, should there be some standard for visits that do and do not count? And if so, what should that standard be? There is insufficient evidence to guide these decisions, but these are empirical questions that we can address in the ZS evaluation project. Once specifications of what counts are determined then the challenge is to operationalize the metrics across different health systems with varying capture and documentation of follow-up visits.

### Challenge 3: Trying to determine whether a safety planning discussion took place between a clinician and a patient, and if so, what actually happened

Finally, many variables of interest represent clinical processes that are not discretely captured and easily retrievable in the EHR or claims data. For example, safety planning is a recommended engagement intervention in ZS. A safety plan recognizes that individuals may have chronic or intermittent suicidal thoughts and, as the name implies, the goal is to prepare for how to respond to those thoughts. Safety planning is intended to be a tangible process that is often recorded on paper by the patient in collaboration with the clinician. As such, it can be difficult to identify when safety planning has occurred because paper plans are not always documented in the EHR. Furthermore, unlike the PHQ-9 or the C-SSRS that have discrete response categories, safety plans are unique to each patient and the majority of safety plan components require an open, unstructured response format. Text is often embedded in clinical progress notes and rarely in a discrete, retrievable field. Therefore, apart from time-intensive manual chart review, it can be difficult to determine whether and how many specific safety plan components (e.g., identification of warning signs, internal coping strategies, distractions, supports) were completed.

To address some of these challenges, all of the participating health systems are working together to build standardized EHR-based safety planning tools. While the health systems have agreed to use a core set of safety planning components (in most cases modeled after the Stanley-Brown template [23]), each requires the ability to implement local adaptations, such as additional questions that their clinicians feel are important to include as well as local crisis resources. Further, it has proven difficult to create a single safety planning template that accounts for differing documentation norms and preferences across health systems. As a result, Kaiser Permanente is building a customizable set of national tools that can be linked to a common set of core safety planning variables. This approach will allow local adaptation of the safety plans such that unique additional site-specific components may be added to the standard components and local resources may be customized. Importantly, this approach allows front-end, clinician-facing safety plan presentations that accommodate documentation preferences within each health system to be yoked to back-end smart data elements (i.e., EHR data entities for capturing discrete values; can be linked to other EHR tools such as smart forms) that are common across the health systems. These common data elements then enable comparisons for research and evaluation. For example, the task of identifying warning signs can be displayed as a “doc flowsheet” in health system A, as a “smart form” in health system B, or as a “smart phrase” in health system C. Each of these different displays is linked to a single smart data element that can easily be identified by quality improvement teams or researchers. To evaluate the impact of safety planning on suicide outcomes in our ZS evaluation project, safety planning exposures (in general and specific components) will be measured using these smart data elements. We will create a binary variable to indicate that a safety plan was invoked during a patient encounter.

The scope of the evaluation project does not include examining the quality of safety plans to better understand what actually happened in the safety planning exchange between the clinician and the patient. However, a subset of a few sites participating in the larger project have received supplemental funding to develop an advanced method using natural language processing (NLP) to determine when lethal means assessment and safety planning have been documented in the narrative, open-text sections of progress notes in the chart or in the electronic safety plan templates linked to smart data elements. Simple programs can give an indication of quality such as whether all sections of the safety plan were completed, whether phone numbers and crisis contacts were provided, or whether a minimum of three coping skills were documented. More advanced methods using NLP could include more sensitive content evaluation such as: whether there was inclusion of proper names of informal contacts, references to feelings or behaviors documented in the warning signs, and whether a plan for lethal means removal was documented when appropriate.

### Major Themes

This paper details the challenges of conducting a large-scale, multi-site suicide prevention evaluation and creating generalizable suicide prevention metrics using health system data. Specifically, we highlight the early experiences of our health systems as they have adopted ZS, demonstrated by three progressively more difficult challenges in measuring process improvements. First, mapping and reconciling suicide risk assessments involves a relatively standard care process (PHQ-9 or C-SSRS) that’s usually recorded as discrete data elements (responses to specific PHQ-9 or C-SSRS questions). There is some variability in PHQ-9 or C-SSRS versions across systems, but the number of variations is finite and relatively small. Then assessing adequate outreach and follow-up involves much more variable care processes (many routes for and types of outreach contacts) and more variability in how contacts are recorded. The number of variations is finite, but much larger. Finally, assessing adequacy of safety plans involves an infinitely variable care process (a highly variable interaction between two human beings) that can be recorded in an infinite number of ways (mostly in free text). This is the most challenging problem to overcome.

To address each of these problems, our team has followed a similar approach. Whenever possible, when we have been engaged early in the cycle of care improvement, we have recommended that health system leaders standardize care processes and their documentation. We have advocated for using standardized screening tools and encouraged that measures be recorded in the EHR (e.g., through use of templates) or be universally retrievable (e.g., through use of smart data elements). Serving as embedded researchers in our local ZS implementation teams and in the multi-system ZS learning collaborative has facilitated this partnership. When care improvement processes have been in place and we could not influence their design, we have engaged first with our clinical partners to identify all the relevant care processes and then with our health system data architects to identify the ways those processes are recorded in the EHR so we are not systematically missing important data.

## Conclusions

The challenges described herein are generalizable beyond suicide prevention work. The challenge of aligning process and outcome measures for comparison across health systems with differing source data is longstanding and universal. The challenge of deciding what should count as adequate and appropriate follow-up care has beset national quality improvement measures when well-intended metrics fail to specify qualifying services or do so in ways that exclude important care processes or include extraneous and irrelevant visits. The challenge of assessing safety planning is similar to the challenge of using EHR records to assess whether shared decision-making about treatment alternatives actually happened—where the presence of an indicator of shared decision-making is probably insufficient to conclude that meaningful patient engagement actually occurred. These are problems that deserve attention. In the context of this pragmatic evaluation, we resolve to address these issues as we seek to create generalizable process improvement metrics for broad use.
